# Budget impact analysis on erythropoiesis–stimulating agents use for the management of chemotherapy-induced anaemia in Greece

**DOI:** 10.1186/1478-7547-11-16

**Published:** 2013-07-16

**Authors:** Eleftheria Nikolaidi, Magdalini Hatzikou, Mary Geitona

**Affiliations:** 1 School of Social Sciences, University of Peloponnese, Corinth, Greece

**Keywords:** Budget impact, Biosimilars, Erythropoiesis Stimulating agents (ESAs), Greece

## Abstract

**Background:**

Chemotherapy-induced anaemia is a common and significant complication of chemotherapy treatment. Blood transfusion and administration of Erythropoiesis-Stimulating Agents (ESAs) either alone or in combination with iron are the most widely used therapeutic options. In Greece, ESAs are among the top ten therapeutic groups with the highest pharmaceutical expenditure, since they are fully reimbursed by social security funds. The objective of the study is to determine potential cost savings related with the use of biosimilar over originator ESAs for the management of the newly diagnosed chemotherapy-induced anemic patients.

**Methods:**

A budget impact analysis has been carried through the elaboration of national epidemiological, clinical and economic data. Epidemiological data derived from WHO (GLOBOCAN) and the European Cancer Anaemia Survey. Clinical data reflect oncology patients’ disease management. ESAs consumption was based on data from the biggest social security fund (IKA). The administration of ESAs under different dosing schemes and time periods has been estimated by separating them in originators and biosimilars as well as by classifying anaemic patients in responders and non-responders. Cost analysis is based on newly diagnosed patients’ alternative treatment scenarios. Treatment costs and prices are used in 2012 values. The Social Security Funds’s perspective was undertaken.

**Results:**

Based on the annual incidence rates, 2.551 newly diagnosed chemotherapy-induced anemic patients are expected to be treated with ESAs. Average cost of treatment on originators ESAs for responders is €2.887 for the 15-week ESAs treatment and €5.019 for non-responders, while on biosimilars €2.623 and €4.009 respectively. Treatment cost on biosimilars is 10.1% lower than originators for responders and 25.2% for non-responders. Budget impact estimates show that treating anemic patients with originator ESAs was estimated at €10.084.800 compared to €8.460.119 when biosimilar ESAs were used, leading to an overall 19,20% cost reduction favoring biosimilars.

**Conclusion:**

In Greece, the treatment on biosimilar ESAs seems to be a cost saving option over originators for the newly diagnosed chemotherapy-induced anemic patients, since it corresponds to 5% of the annual overall consumption and expands patients’ access to ESAs treatment. Health care decision making should rely on evidence based treatments in order to achieve social funds’ sustainability in an era of economic recession.

## Introduction

The European Cancer Anaemia Survey (ECAS) has revealed a high prevalence and incidence rates of anaemia in cancer patients in Europe since it examined disease’s evolution, severity and management in a large and representative population sample of European cancer patients [1,2]. Chemotherapy-induced anaemia is a common and significant complication of chemotherapy treatment, with an incidence rate of 62.7% [1]. Additionally, anaemia has been reported in 19.5% of patients in the first chemotherapy cycle rising up to 46.7% of patients in the fourth and fifth chemotherapy cycle [1].

Therapeutic options provide supportive care through transfusion with packed red blood cells or administration of Erythropoiesis-Stimulating Agents (ESAs), with or without iron supplementation [3,4]. Transfusions are recommended to correct very low (<9 g/dl) Hb levels, but not for Hb values above that level [4-6]. Additionally, they are related with risk issues such as infections, disease transmissions and blood availability reduction [4,7-10].

Supplementing ESA therapy with intravenous iron has been shown to increase hemoglobin response rates compared with ESA alone, to levels similar to those observed for transfusions [11-13]. This evidence is also supported by Aapro et al., 2012 [14] review which showed higher efficacy of intravenous iron over oral or no iron in reducing blood transfusions, increasing haemoglobin levels, and improving quality of life in ESAs-treated anaemic cancer patients. Additionally, by adding intravenous iron to ESA therapy the cost of ESA treatment across the therapeutic class is reduced [15].

ESAs are genetically engineered forms of erythropoietin. Based on their approved indications, by the European Medicines Agency (EMA), they are used for the treatment of symptomatic anaemia associated with chronic renal failure as well as the treatment of adult cancer symptomatic anaemia patients with non-myeloid malignancies receiving chemotherapy. It should be mentioned that there is a controversy in the international literature concerning safety and effectiveness issues related with the use of ESAs [16,17].

There is suggestive evidence that the use of ESAs in patients with chemotherapy-induced anaemia may improve disease-specific measures of quality of life and decreases the use of blood transfusions [18-25].

The first biosimilars of ESAs were developed after the patent expiry of the originators. The term biosimilar designates a second-entry version of biological medicinal products whose complex molecular structure is more challenging and requires greater resources than traditional generics [26-31]. The approval process that has been applied for generics is unsuitable for biosimilars and specific provisions for these products were developed. Their authorization is granted on the basis of quality, safety and efficacy data [32]. There is a real choice available to physicians when selecting different ESAs products, since it appears to have no significant difference in the effectiveness and safety of different agents in managing chemotherapy-induced anaemia [5,33].

ESAs are among the top ten therapeutic groups with the highest pharmaceutical expenditure ranging from 26,4% in 2008 to 22,7% in 2010, as reported by IKA, the biggest social security fund in Greece until 2011 [34]. The market share (MS) of originator epoetins was 99% in 2008 corresponding to €41,4 million. In 2009 ESAs expenditure reached €43 million, with 94% and 6% MS for original and biosimilar ESAs respectively, whilst in 2010 an inverse tendency appeared presenting an increase of MS of biosimilars to 19%. The same year ESAs expenditure was almost halved (€19 million) since cost-containment measures were implemented such as flat price cuts and stricter control of prescriptions [35]. Therefore, although ESAs are widely used treatments, they remain a costly option since they are fully reimbursed (100%) by all social security funds without any patient co-payment. Under the current economic recession that Greece is facing, numerous cost containment measures have been implemented in all fiscal sectors. In the health care sector, the majority of the measures performed targeted to pharmaceutical expenditure [35].

The objective of this paper is to elaborate epidemiological, clinical and economic data on ESAs consumption in Greece, in order to determine potential cost savings by using biosimilar over originator ESAs under different dosing schemes for the management of the newly diagnosed chemotherapy-induced anemic patients.

## Methods

A budget impact analysis has been carried out based on national epidemiological, clinical and economic data. Epidemiological data concerning the incidence of cancer in Greece derived from IARC-WHO (GLOBOCAN) 2008 [36]. Anaemia treatments separated in ESAs (either alone or in combination with iron and/or transfusion), transfusion (alone or with iron) and iron alone were based on the European Cancer Anaemia Survey (ECAS) [1]. Incidence of cancer–associated anaemia for the Greek oncologic patients was performed by type of cancer: breast, lung, GI/colocteral, head/neck, gynecological, lymphoma/myeloma, leukemia, urogenital and a category containing the remaining cancer types.

The classification of ESAs per Hb level and strength has been estimated by separating them in originators (darbepoetin α, epoetin α and epoetin β) and biosimilars (epoetin α and epoetin ζ) for a two month period as well as for a time period of six chemotherapy cycles on ESA treatment, aligned with routine chemotherapy duration. Approved ESAs classification, per strength and price among originators and biosimilars marketed in Greece is presented in Table 1.

**Table 1:** **ESAs classification per strength (IU/mcg) and price between originator** &**biosimilar products in 2012**

**Originators**			
**Brand**	**Strength**	**Active substance**	Average hospital price/syringe (€)*
	150 MCG		192.27
ARANESP	300 MCG	Darbepoetin α	385.11
	500 MCG		635.49
	10.000 IU		60.79
NEO RECORMON	20.000 IU	Epoetin β	149.68
	30.000 IU		190.16
	60.000 IU		342.25
	10.000 IU		53.9
EPREX	20.000 IU	Epoetin α	122.79
	30.000 IU		196.55
	40.000 IU		212.42
**Biosimilars**			
**Brand**	**Strength**	**Active substance**	Average hospital price/syringe (€)*
ABSEAMED	10.000 IU		50.26
	20.000 IU	Epoetin α	101.91
	30.000 IU		152.87
	40.000 IU		203.83
	10.000 IU		47.41
BINOCRIT	20.000 IU	Epoetin α	94.37
	30.000 IU		141.55
	40.000 IU		188.73
	10.000 IU	Epoetin ζ	54.51
RETACRIT	40.000 IU		255.54

*Hospital Prices 2012.

The recommended initial doses of ESAs, based on their approved by EMA Summary of Product Characteristics (SPC) are 30.000 IU/week (or 3 × 10.000 IU/week) for epoetin α, epoetin β and epoetin ζ and 150 mcg/week for darbepoetin α. Alternate approved doses for epoetin α/epoetin ζ and darbepoetin α are 40.000 IU/week and 500m cg/3 weeks respectively.

If Hb has not increased by at least 1 g/dl after 4-week treatment with epoetin α, epoetin β and epoetin ζ, the dose may be escalated to 200% of the initial dose. Based on the US National Comprehensive Cancer Network, (NCCN) clinical practice guidelines in Oncology for the treatment of cancer and chemotherapy-induced anaemia [37], the dose of darbepoetin α could be increased up to 4.5 μg/kg once a week corresponding to 300 μcg/week in case of no response at 6-week treatment with the initial darbepoetin dose. In order to be able to compare darbepoetin with the rest epoetins, at various stages of the chemotherapy-induced anaemia treatment, the escalation of dosage scheme to 200% was performed in 4 weeks interval instead of 6 weeks (Table 2).

**Table 2:** Dosage Scheme of ESAs for “Responders” and “Non-Responders” Anaemic Patients for a two-month period

**Active substance**	**Responders (Hb increase ≥ 1 g/dl)**		**Non responders (Hb increase < 1 g/dl)**	
	**First month treatment**	**Second month treatment**	**First month treatment**	**Second month treatment**
Darbepoetin α	150 MCG/week or 500 MCG/3 weeks	150 MCG/week or 500 MCG/3 weeks	150 MCG/week or 500 MCG/3 weeks	300 MCG/week
Epoetin β	3 × 10.000 IU/week or 30.000 IU/week	3 × 10.000 IU/week or 30.000 IU/week	3 × 10.000 IU/week or 30.000 IU/week	3 × 20.000 IU/week or 2 × 30.000 IU/week or 1x60.000 IU/week
Epoetin α	3 × 10.000 IU/week or 40.000 IU/week or 30.000 IU/week	3 × 10.000 IU/week or 40.000 IU/week or 30.000 IU/week	3 × 10.000 IU/week or 40.000 IU/week or 30.000 IU/week	3 × 20.000 IU/week or 2 × 30.000 IU/week
Epoetin ζ	3 × 10.000 IU/week or 40.000 IU/week	3 × 10.000 IU/week or 40.000 IU/week	3 × 10.000 IU/week or 40.000 IU/week	6 × 10.000 IU/week

In case the Hb has not increased by at least 1 g/dl after 4 consecutive weeks (8 weeks in total) with the escalated ESAs dose, the treatment with epoetin α, epoetin β and epoetin ζ should be discontinued. Concerning darbepoetin α, in case of patient’s non-response (fatigue, haemoglobin response) after nine weeks of treatment, further therapy may not be effective, according to the SPC recommendations.

Based on the national clinical guidelines [38] as well as the European Society for Medical Oncology (ESMO) guidelines [39], chemotherapy duration varies from 4–6 cycles depending on the type of cancer. In the current analysis six cycles of chemotherapy were used at three-week intervals. A chemotherapy cycle was defined as the three-week period with the chemotherapy being administered at the first week of each cycle. The 6-cycle chemotherapy corresponds to 18 weeks. The initiation of ESAs treatment was assumed to take place at the beginning of the second chemotherapy cycle i.e. at week 4 and continued until the end of the 6th cycle of chemotherapy (end of week 18), for a total of 15 weeks [1,5].

Two different ESAs escalating dosing schemes were used (Table 2) leading to the classification of chemotherapy-induced anaemic patients in responders and non-responders with a 50% response rate per group. Responders were administered the recommended initial doses of ESAs for the total of 15 weeks, while non responders’ dosing scheme was escalated to 200% of the initial dose in the beginning of the week 9 until the end of 15th week.

Each ESA can be used in more than one fixed dosing scenario (Table 2). In order to enable a clinically relevant comparison, the cost of all possible fixed dosing schemes were taken into account and average cost estimates per month of originator and biosimilar ESAs were used.

Cost analysis among originator and biosimilar ESAs was based on a two-month treatment period per patient. In the budget impact analysis the cost estimates for the two-month period treatment were extrapolated to a six-cycle chemotherapy framework based on the incidence rates, Hb level, distributed in responders and non responders. Hospital prices were averaged using 2012 values. The Social Security Funds’s perspective was undertaken and for data elaboration Microsoft Excel version 7 was used.

## Results

Newly diagnosed cancer patients in Greece are estimated at 37.089 out of which 14.660 are expected to be treated for anaemia (Table 3). From those 2.551 (17.4%) chemotherapy-induced anaemic patients will be treated with ESAs. Transfusion alone or with iron, was the second most frequent choice of physicians administered to 2.184 patients (14.9%) and iron alone was the last one administered to 953 patients (6.5%).

**Table 3:** Incidence of patients with cancer associated anaemia in Greece and categorization per type of anaemia treatment

**Type of cancer**	**% Of patients with cancer associated anaemia on treatment**^**1**^	**Newly diagnosed cancer patients in Greece**^**2**^	**Number of Greek patients with cancer associated anaemia or treatment**^**1,2**^	**Number of Greek patients on treatment with ESAs (17,4%)**^**1,2**^	**Number of Greek patients on treatment with transfusion (14,9%)**^**1,2**^	**Number of Greek patients on treatment with iron only (6,5%)**^**1,2**^	**Number of Greek patients under no treatment (61,2%)**^**1,2**^
Breast	26.2	4.349	1.139	198	170	74	697
Lung	47.7	6.667	3.180	553	474	207	1.946
Gl-Colon	33	5.062	1.670	291	249	109	1.022
Head & Neck	46.1	1.100	507	88	76	33	310
Gynaecological	42.7	2.026	865	151	129	56	529
Lymphoma/myeloma	47.4	1.388	658	114	98	43	403
Leukaemia	53.3	1.512	806	140	120	52	493
Urogenital	43	5.610	2.412	420	359	157	1.476
Other	36.5	9.375	3.422	595	510	222	2.094
Total	375.9	37.089	14.660	2.551	2.184	953	8.972

Sources: ^1^(Ludwig et al. 2004), ^2^WHO-Globocan 2008.

The cost of ESAs treatment classified per Hb level, responders and non responders on originators and biosimilar, is presented in Table 4. The cost difference for responders on originators versus biosimilars is €336.511 and for non-responders is €1.288.170 respectively. The savings associated with the use of biosimilars for both patients categories (responders and non-responders) is estimated at €1.624.681.

**Table 4:** **Cost of 15-week ESAs treatment (6-cycles chemotherapy) for responder** &**non responder patients receiving originators or biosimilars per Hb level**

			**Responders**	**Non responders**	**Responders**	**Non responders**	**Cost difference for responders originators vs. Biosimilars (€)**	**Cost difference for non responders originators vs. Biosimilars (€)**	**Total difference originators vs. Biosimilars (€)**
**HB Nadir (g/dl)**	**% of patients per Hb level**	**Number of Greek patients treated with ESAs per Hb Level**	**Cost of 15 weeks treatment with originator ESAs (€)**	**Cost of 15 weeks treatment with originator ESAs (€)**	**Cost of 15 weeks treatment with Biosimilar ESAs (€)**	**Cost of 15 weeks treatment with Biosimilar ESAs (€)**			
<9	33.5	855	1.233.547	2.144.861	1.120.816	1.713.324	112.731	431.537	544.268
9.0-9.9	27.5	701	1.012.613	1.760.707	920.072	1.406.460	92.541	354.247	446.787
10.0-10.9	24.7	630	909.511	1.581.435	826.392	1.263.257	83.118	318.178	401.296
11.0-11.9	9.5	242	349.812	608.244	317.843	485.868	31.969	122.376	154.345
>12.0	4.8	122	176.747	307.323	160.594	245.491	16.153	61.832	77.985
**Total**	**100**	**2.551**	**3.682.229**	**6.402.570**	**3.345.718**	**5.114.400**	**336.511**	**1.288.170**	**1.624.681**

Average cost per patient for responders is € 2.887, using originators (darbepoetin α, epoetin α and epoetin β) for the 15-week ESAs treatment (6-cycles chemotherapy) and € 5.019 in the case of non-responders. Average cost of patients on biosimilar treatment (epoetin α and epoetin ζ) is € 2.623 for responders and € 4.009 for non-responders. Responder patients on biosimilars were estimated to have 10.1% lower cost than patients on original ESAs. In case of non-responders, the cost difference between originators and biosimilars is even higher reaching approximately 25.2% favoring also biosimilars.

## Discussion

Budget impact estimates reveal that treating newly diagnosed chemotherapy-induced anemic patients with originator ESAs for 6 cycles chemotherapy was estimated at €10.084.800 compared to €8.460.119 when biosimilar ESAs were used in both responder and non-responder patient categories, leading to an overall 19,20% cost reduction favoring biosimilars. Consequently, budget impact analysis showed that the administration of biosimilar ESAs seems to be a cost saving option over originators, since the savings from the use of biosimilars approximates 5% (€1,5million) of the total annual ESAs consumption in the country. It should be noted that the above mentioned savings correspond to the additional treatment of 490 anaemic patients.

However, there are certain limitations in the current analysis that should be discussed. First, given the limited experience in the use of biosimilars, it is rather difficult to compare and generalize our findings with international literature. The only study with comparable results is that of Aapro et al. [5] which compared the cost of chemotherapy-induced anaemia among originator epoetin α, epoetin β and darbepoetin α and biosimilar epoetin α at the level of a single patient using various dosing scenarios. Although the above study compared a single biosimilar agent with the rest originators category whilst in our analysis all biosimilar treatments were used and compared with the whole originators category, still both studies revealed saving by the use of biosimilars. Second, given that substitution on ESAs is not recommended by the European Agencies for medicines, the current research hypothesis is conservative, since it is based only on incidence rates without taking into consideration cancer patients already on treatment with an originator ESA. Additionally, the rates of cancer anaemic patients on treatment were based on ECAS study performed in 2004, which might not reflect potential changes in the current epidemiological rates. Also the assumption of 50% response rate of the cancer anaemia patients was necessary due to lack of respective international data in order to keep a balance between the comparable categories. Finally, the fact that the present study focuses on the ESAs consumption and does not take into consideration neither the alternative treatment option of transfusions nor the supplementary treatment of ESAs with iron constitutes another limitation.

It is believed that although sound national and international evidence have been used for the estimation of epidemiological chemotherapy-induced anaemia and disease management data, still further research on patient reported outcomes deriving from empirical evidence, is needed. In addition, the authors would like to point out that an evaluation of the efficacy/effectiveness and safety of ESAs for their intended use and their approved therapeutic indications were not within the aims of the study. The current budget-impact analysis was based on the use of ESAs in Greece, their approved therapeutic indications and posology regimens, among originators and biosimilars.

Despite the above research limitations, it should be mentioned that the conduction of budget impact analysis of originators versus biosimilars seems to be important both at the micro and macro-economic level, especially for reimbursement decisions making and health resources re-allocation purposes. However, comparing among alternative therapeutic options for managing chemotherapy-induced anaemia, more complex analysis is needed. The conduction of cost-effectiveness or cost-utility analyses focusing on alternative management strategies for chemotherapy-induced anaemia might result in more comparable treatment costs and clinical outcomes. Economic evaluation studies combine budgets’ savings with clinical evidence that could lead in the expansion of patients’ access to ESAs, the avoidance of blood wastes as well as to the improvement of patients’ quality of life.

## Conclusion

The administration of biosimilar ESAs to newly diagnosed chemotherapy-induced anemic patients seems to be a cost saving option over originators, although further research on patient reported outcomes and empirical evidence is needed. Under the economic recession that Greece is experiencing, health care decision making should rely on evidence based treatments in order to achieve social funds’ sustainability.

## Abbreviations

ESAs: Erythropoiesis-Stimulating Agents; Hb: Heamoglobin; ECAS: European Cancer Anaemia Survey; EMA: European Medicines Agency; MS: Market Share; IKA: Social Security Fund; SPC: Summary of Product Characteristics; NCCN: US National Comprehensive Cancer Network; ESMO: European Society for Medical Oncology.

## Competing interests

The authors declare that they have no competing interests.

## Authors’ contributions

All authors have equally contributed to the writing of the manuscript and MG critically reviewed, corrected and approved the final version.

## Authors’ information

MG is Associate Professor of Health Economics at University of Peloponnese. MH is research associate at University of Peloponnese and holds the position of Health Economics Manager at Novartis Hellas. EN is postgraduate student at University of Peloponnese and employee of National Organization for Medicines in Greece.
